# 3.Q. Workshop: Using foresight to anticipate future public health challenges

**DOI:** 10.1093/eurpub/ckac129.198

**Published:** 2022-10-25

**Authors:** Organised by: RIVM (Netherlands), Sciensano (Belgium), PHIRI, EUPHA-PHMR, EUPHA-FS, Chair persons: Henk Hilderink (EUPHA-FS), Petronille Bogaert (EUPHA-PHMR)

## Abstract

The COVID-19 pandemic brought great uncertainty about current and future health impacts. Uncertainty about how the virus would directly impact our health. And uncertainty about indirect impacts of COVID-19 on long-term health and wellbeing, including consequences due to delayed prevention, diagnosis, and medical treatment. At this point in time, we know a lot more about the immediate mortality and morbidity aspects. How we fare on the wider impacts on the longer term is more difficult to say. A structured tool for gaining in sight in this, is foresight. We use this tool within the Population Health Information Research Infrastructure (PHIRI). PHIRI supports research across Europe through the identification, access, assessment and reuse of population health and non-health data to underpin public health policy decisions. PHIRI is a practical use case and lays the foundation for ultimately developing a Distributed Infrastructure on Population Health (DIPoH). The joint workshop aims to show ways to better understand the complexity of the wider impacts of the SARS-COV-2 virus on population health across Europe; show how a research infrastructure might help researchers in Europe build capacity and helping each other to achieve higher goals. The workshop will begin with a presentation providing a brief overview of the PHIRI foresight work. This will be followed by two researchers who participated in the PHIRI foresight training program and undertook their own Public Health Foresight study. In the last presentation of the session foresight will be reflected on from a policy perspective. How can researchers and health policy makers better interact to have better foresight-informed policy making. Interaction with the audience will be guaranteed using Mentimeter questions. With this interactive element, insights art obtained what people find the most important trends to act upon, what they consider most important future challenges and how researchers and policymakers can better interact.

**Key messages:**

• Foresight studies are essential to be better prepared for and to anticipate to future challenges.

• With foresight-informed policy making direct and indirect health impacts of COVID-19 are addressed.

